# Complete Genome Sequence of *Pseudomonas* sp. Strain BIOMIG1^BAC^, Which Mineralizes Benzalkonium Chloride Disinfectants

**DOI:** 10.1128/MRA.00309-20

**Published:** 2020-05-14

**Authors:** Recep Can Altinbag, Emine Ertekin, Ulas Tezel

**Affiliations:** a Institute of Environmental Sciences, Bogazici University, Istanbul, Turkey; University of Arizona

## Abstract

*Pseudomonas* sp. strain BIOMIG1^BAC^ is an antibiotic-resistant gammaproteobacterium that can completely mineralize different homologs of benzalkonium chloride disinfectants. Here, we report the annotated complete genome sequence of this microorganism, which includes one circular chromosome with a length of 7,675,262 bp.

## ANNOUNCEMENT

*Pseudomonas* sp. strain BIOMIG1^BAC^ was isolated from a sample taken from the influent of a Pasakoy urban wastewater treatment plant located in Istanbul, Turkey, after enrichment in a salt medium containing benzalkonium chlorides (BACs) as the sole carbon and energy source, followed by selective plating on CHROMagar *Pseudomonas* (1). Strain BIOMIG1^BAC^ can convert BACs to ammonium through a sequence of dealkylation, debenzylation, and demethylation reactions; it is the first BAC degrader that has been fully characterized metabolically and genetically (2). Strain BIOMIG1^BAC^ is not only tolerant to cationic disinfectants but also resistant to a broad range of antibiotics, including beta-lactams, sulfonamides, macrolides, and glycopeptides, mainly through multidrug efflux pumps.

Relatively recently, the draft genome of BIOMIG1^BAC^ was published (GenBank accession number MCRS00000000) (2). However, based on the universal single-copy essential genes, the draft genome of BIOMIG1^BAC^ was only 92.8% complete. In order to obtain the complete genome sequence of strain BIOMIG1^BAC^, we concurrently used short reads obtained from the Illumina HiSeq 2000 platform and long reads generated with the Oxford Nanopore Technologies (ONT; Oxford, England) MinION platform. We used the short reads obtained in our previous study (2). In order to obtain those shotgun short-read sequences, the genomic DNA of strain BIOMIG1^BAC^ was isolated from an overnight culture initiated from a single colony of the strain by using the NucleoSpin Soil DNA extraction kit (Macherey-Nagel, France). DNA was processed with the Illumina TruSeq DNA PCR-free library preparation kit and sequenced using the Illumina HiSeq 2000 platform in the next-generation sequencing department of Macrogen, Inc. (Seoul, South Korea). The Illumina HiSeq 2000 platform generated 10,757,826 paired-end short reads, with total nucleotides equal to approximately 1.1 Gb. The genomic DNA used in shotgun sequencing with the MinION platform was isolated from BIOMIG1^BAC^ cells that had been cultivated for 2 days with 200 mg/liter BAC by using EZ-DNA reagent (Bio Basic, Ontario, Canada), followed by ethanol precipitation and affinity column purification (Macherey-Nagel). About 400 ng DNA was treated with a rapid sequencing kit (SQK-RAD004; ONT) and loaded into a FLO-MIN106 flow cell (flow cell identification number FAL49576) after priming of the flow cell following the protocols described for the kit. The MinION platform generated 2,681,861 long reads that summed to 8.5 Gb of total nucleotides, with an average length of approximately 30 kb (3). The raw signal file generated from the MinION run was converted to a fastq file with Guppy v3.4.4 base caller software (ONT) (4) using dna_r9.4.1_450bps_fast.cfg as the configuration file. After creation of the fastq file, Filtlong software was used to eliminate reads with a score below Q10. Unicycler was used to combine short and long reads to obtain the whole genome of BIOMIG1^BAC^ (5). Unicycler first cleaned the raw short reads and created a SPAdes assembly with a k-mer size of 67, 618 contigs, and 3 dead ends; it then combined those reads with the long reads to construct the whole genome (6). In the end, a circular genome with a length of 7,675,262 bp was obtained. The genome was annotated with the NCBI Prokaryotic Genome Annotation Pipeline (PGAP) using default settings (7). In summary, the genome of BIOMIG1^BAC^ contains 7,146 protein-coding sequences, 7,238 genes, 7 regulatory sequences, 1 CRISPR sequence, 5 16S rRNA genes, and 72 tRNA genes. The G+C content of the genome is 62.1%. A gene located between the 6,844,288th and 6,845,439th bases in the complete genome encodes a novel Rieske oxygenase, oxyBAC (2), which transforms BACs to benzyldimethylamine. The complete genome has about 500 genes more than the draft genome. In particular, the numbers of insertion sequences (ISs), including transposons and integrases, are significantly greater in the complete genome than in the draft genome. For instance, the complete genome has 104 ISs and 43 integrases, whereas the draft genome has only 29 ISs and 29 integrases. Longer reads facilitated the identification of mobile genetic elements and the associated genes in the genome, as mentioned in other studies (8). The genome size of strain BIOMIG1^BAC^ is relatively large for a *Pseudomonas* species. Such a large genome with large numbers of ISs and integrases suggests that BIOMIG1^BAC^ has an elastic genome that may contain other novel catabolic functions open to discovery.

### Data availability.

The complete genome sequence of *Pseudomonas* sp. strain BIOMIG1^BAC^ has been deposited at DDBJ/ENA/GenBank under the accession number CP049045. The raw reads obtained from the Illumina HiSeq 2000 and MinION platforms are available in the NCBI database under the accession numbers SRX7964272 and SRX7964273, respectively.
